# Overexpression of *OsMed16* Inhibits the Growth of Rice and Causes Spontaneous Cell Death

**DOI:** 10.3390/genes12050656

**Published:** 2021-04-27

**Authors:** Jie Jiang, Guangzhe Yang, Yafeng Xin, Zhigang Wang, Wei Yan, Zhufeng Chen, Xiaoyan Tang, Jixing Xia

**Affiliations:** 1 State Key Laboratory for Conservation and Utilization of Subtropical Agro-Bioresources, College of Life Science and Technology, Guangxi University, Nanning 530004, China; 1508405003@st.gxu.edu.cn (J.J.); 20150061@gxu.edu.cn (G.Y.); 1908301066@st.gxu.edu.cn (Y.X.); 1808401019@st.gxu.edu.cn (Z.W.); 2 Guangdong Provincial Key Laboratory of Biotechnology for Plant Development, School of Life Sciences, South China Normal University, Guangzhou 510631, China; yanwei_bio@126.com; 3 Shenzhen Institute of Molecular Crop Design, Shenzhen 518107, China; zhufengchen@163.com; 4 Shenzhen Agricultural Technology Promotion Center, Shenzhen 518055, China

**Keywords:** Mediator subunit, *OsMed16*, *Oryza sativa*, spontaneous cell death, defense response

## Abstract

The Mediator complex transduces information from the DNA-bound transcription factors to the RNA polymerase II transcriptional machinery. Research on plant Mediator subunits has primarily been performed in Arabidopsis, while very few of them have been functionally characterized in rice. In this study, the rice Mediator subunit 16, *OsMed16,* was examined. *OsMed16* encodes a putative protein of 1301 amino acids, which is longer than the version previously reported. It was expressed in various rice organs and localized to the nucleus. The knockout of *OsMed16* resulted in rice seedling lethality. Its overexpression led to the retardation of rice growth, low yield, and spontaneous cell death in the leaf blade and sheath. RNA sequencing suggested that the overexpression of *OsMed16* altered the expression of a large number of genes. Among them, the upregulation of some defense-related genes was verified. *OsMed16* can regulate the expression of a wealth of genes, and alterations in its expression have a profound impact on plant growth, development, and defense responses in rice.

## 1. Introduction

Unlike prokaryotic genes, transcription of eukaryotic genes is orchestrated by RNA polymerase II (Pol II) and multiple regulatory proteins, including general transcription factors (TFs), gene-specific TFs, and Mediator [1,2]. Mediator is a highly conserved multiprotein complex that consists of 25–34 subunits depending on the species [3]. The structure of the whole Mediator complex can be divided into three main modules (head, middle, and tail) and a transiently associated kinase module, and every module has different functions in transcription [3]. The head and middle modules constitute the core Mediator and contact Pol II and general TFs, while the tail module interacts with gene-specific TFs [3,4]. The kinase module and Pol II associate with the main modules in a mutually exclusive fashion; therefore, they act as transcriptional repressors [5,6]. Generally, during the formation of Pol II preinitiation complex (PIC), Mediator can transmit regulatory information from DNA-bound TFs to the basal transcriptional machinery, thereby regulating the expression of downstream genes [3,4].

Mediator was first identified biochemically in yeast in 1990 [7], and its counterparts were subsequently isolated from humans and other animals [8,9,10]. The biochemical identification of the Mediator complex in plants took place much later. The first plant Mediator complex was purified from an Arabidopsis cell suspension culture in 2007 [11]. In addition to multiple Mediator subunits, Pol II subunits were also isolated in the purified Arabidopsis Mediator fraction, but the kinase module subunits (Med12, Med13, CDK8, and CycC) were not isolated together with the bulk complex. Furthermore, the kinase module subunits were identified by bioinformatics approaches [11]. Currently, the Arabidopsis Mediator complex is commonly thought to comprise 33 subunits, including 29 subunits that are conserved with their yeast or animal counterparts and four subunits that are unique to plants [3,12]. To date, Mediator subunits in other plants have not yet been biochemically identified, but they have been characterized by bioinformatics analyses. Mathur et al. [13] identified Mediator subunits in silico in 16 plant species ranging from algae to higher angiosperms. It was determined that at least one homolog for all the animal/fungal Mediator subunits is present in the plant kingdom. In addition to in silico analysis, the biological functions of some Arabidopsis Mediator subunits have been studied through genetic and molecular analyses. It was found that these Mediator subunits participate in multiple biological processes, including plant growth, development, flowering, pathogen defense, and stress tolerance [14,15,16,17,18].

Rice is an important staple crop, which is also used as a model plant for monocots. A total of 55 Mediator genes, including paralogs of some main module subunits and kinase module subunits, have been identified in the whole rice genome by in silico approaches [13]. However, unlike the situation in Arabidopsis, very few rice Mediator subunits have been functionally characterized. *OsMed15a* and *OsMed14-1* are the two well-studied Mediator subunits in rice. *OsMED15a* is implicated in rice seed development through the linking of rice grain size/weight-regulating TFs to their target genes. A reduction in the expression of *OsMed15a* in RNAi plants downregulated the expression of genes associated with grain size/weight, *GW2*, *GW5*, and *DR11*, and reduced the grain length, weight, and yield [19]. *OsMed14-1* plays an important role in rice development. The RNAi-mediated repression of the expression of *OsMed14-1* led to growth inhibition and slender organs, which was caused by defective cell-cycle progression and reduced the level of auxin in *OsMed14-1* knockdown plants [20].

*OsMed16* (*OsSFR6*) is a homolog of *AtSFR6*, and its function has been preliminarily studied in Arabidopsis [21]. The *atsfr6* mutant was sensitive to freezing and had pale cotyledons and leaves. The overexpression of *OsMed16* in the *atsfr6* mutant could restore the wild type phenotype and elevate its tolerance to freezing and osmotic stress [21]. Moreover, the expression of *COLD-ON REGULATED* (*COR*) genes could also be restored in an *atsfr6* mutant that overexpressed *OsMed16*; thus, *OsMed16* is thought to act as a regulator of COR gene expression, osmotic stress, and freezing tolerance in Arabidopsis [21]. However, the biological function of *OsMed16* remains unclear in rice. In this study, the pattern of expression and function of *OsMed16* was investigated in rice. The results revealed that the growth of knockout mutant of *osmed16* was severely inhibited, and the plants were unable to complete their life cycles. The overexpression of *OsMed16* also led to the inhibition of growth, low yield, and spontaneous cell death. RNA-Seq data indicated that the overexpression of *OsMed16* altered the expression of a large number of genes involved in multiple biological processes. In particular, the alterations of some genes related to defense were examined in more detail.

## 2. Materials and Methods

### 2.1. The Plant Materials and Growth Conditions

Wild type rice (*Oryza sativa* cv. Nipponbare), two knockout lines of *OsMed16*, and two *OsMed16* overexpression lines were used in this study. The wild type rice was obtained from the rice resources conservation center of Guangxi University. The *osmed16* mutants were constructed using the CRISPR-Cas9 gene-editing technology in our laboratory (see below). The *OsMed16* overexpression lines were also constructed using Agrobacterium-mediated transformation technology in our laboratory (see below). The cultivation of plants conforms to China’s legislation on genetically modified plants.

The seeds were soaked in deionized water in the dark for 2 days in an incubator at 28 °C. After germination, the seeds were grown either hydroponically or in a paddy field. For hydroponic culture, the seeds were first grown in a solution of 0.5 mM CaCl_2_ for 5–7 days. The seedlings were then transferred to a 4 L plastic pot containing 1/2 Kimura B solution (pH 5.6) [22]. The nutrient solution was changed with a fresh solution every other day. The plants were grown in a greenhouse under natural light at 25–30 °C. The paddy field is located in the rice planting base of Guangxi University, Nanning City, Guangxi Province, China. Each experiment had at least three biological replicates.

### 2.2. Generation of Transgenic Plants

To create the knockout lines of *OsMed16*, the CRISPR/Cas9 genome targeting system was used. The pCRISPR-OsMed16 plasmids with *OsMed16-*specific target sites were constructed as previously described [23]. Briefly, specific target sequences (ATGCCCTCGTGCATTACTGG and GTTGCTTTTGATCCCACTCG) within the *OsMed16* gene were selected by a BLAST search (http://blast.ncbi.nlm.nih.gov/Blast.cgi, assessed on 30 May 2016) of the rice genome sequence. The two specific sequences of the *OsMed16* gene were then, respectively, introduced into the sgRNA expression box by overlapping PCR to produce pU6a-*OsMed16*-SgRNA and pU6b-*OsMed16*-SgRNA.

These fragments were cloned into pYLCRISPR/Cas9 Pubi to construct pCRISPR-*OsMed16* using the restricted connection reactions that contained *Bsa*I and T4 DNA ligase. The constructed plasmids were introduced into *A. tumefaciens* strain EHA101 and transformed into wild type Nipponbare rice. Transformants were selected with hygromycin. The mutants were screened by PCR using primer pairs flanking the *OsMed16*-specific target site, and the homozygous mutants were selected for further study and analysis.

Transgenic plants that overexpressed the *OsMed16* gene (named *OsMed16*-OE) were obtained using *Agrobacterium*-mediated transformation. Total RNA was extracted from Nipponbare using a TRIzol reagent kit (Life Technologies, Carlsbad, CA, USA) and reverse transcribed using a HiScript II Q RT SuperMix Kit (Vazyme, Nanjing, China). The resulting cDNA was used as a template for PCR amplification of the *OsMed16* full length cDNA with 5′-AATTGGTACCATGACCTCTTCCTCCGCCCC-3′ and 5′-AATTACGCGTTCAAACGACTTTCACCCATG-3′ as primers. The full-length cDNA of *OsMed16* was inserted into the pCAMBIA1300-Ubi vector carrying the maize ubiquitin promoter and terminator of the nopaline synthase gene. *OsMed16* gene-specific primers (5′-CGATGGCAATTACACTGTGC-3′ and 5′-TAGAAGGCCAGCAGCATCA-3′) were used to identify the positive transgenic plants. The relative levels of expression of *OsMed16* in transgenic plant leaves were determined by qRT-PCR as described below.

### 2.3. RNA Isolation and Gene Expression Analysis

To examine the expression pattern of the *OsMed16* gene, the roots, leaf blades and sheaths, and spikes were sampled at the heading stage. Total RNA was extracted using Trizol (Thermo Fisher Scientific, Waltham, MA, USA), followed by RNase-free DNaseI treatment. RNA samples were adjusted to 200 ng/μL. For each sample, 1 μg of RNA was used for first-strand cDNA synthesis using a PrimeScript II 1st Strand cDNA Synthesis Kit (TaKaRa, Dalian, China) according to the manufacturer’s instructions. qRT–PCR was performed with ChanQTM SYBR Color qPCR Master Mix (Vazyme, Nanjing, China) on a StepOnePlus Real-Time PCR System (AnalytikJena AG, Jena, Germany) following the manufacturer’s instructions. Three biological replicates (in separated tubes) were performed. The reaction volumes for reverse transcription and PCR were 20 μL. The primers used in analysis of gene expression of *OsMed16* included 5′-CGATGGCAATTACACTGTGC-3′ and 5′-TAGAAGGCCAGCAGCATCA-3′. *Histone H3* was used as an internal standard with the primers 5′-GGTCAACTTGTTGATTCCCCTCT-3′ and 5′-AACCGCAAAATCCAAAGAACG-3′ [24]. Sizes of PCR product are 103 bp for *OsMed16* and 155 bp for *Histone H3*. The relative levels of expression of the genes were calculated using the 2^−ΔΔCT^ method [25], which was carried out using the qPCRsoft3.2 software provided by the manufacturer. The primers for the defense-related genes are shown in Table S1.

### 2.4. Subcellular Localization of OsMed16

To detect the subcellular localization of OsMed16, a plasmid that expressed the OsMed16-GFP fusion protein was constructed. *OsMed16* cDNA was amplified from the Nipponbare cDNA by PCR using the *OsMed16*-specific primers 5′-CCG*GAATTC*ATGACCTCTTCCTCCGCCCC-3′ (*Eco*RI site in italic text) and 5′-CGG*GGTACC*CAACGACTTTCACCCATGTCC-3′ (*Kpn*I site in italic text). The amplified cDNA was cloned downstream of the green fluorescent protein coding region in the PYL322-GFP vector [26] to produce the *OsMed16-GFP* vector.

The vectors expressing the nuclear marker OsGhd7-mcherry, the OsMed16-GFP fusion protein, and GFP alone were all transduced into rice protoplasts. The preparation of rice protoplasts and plasmid transformation has been described previously [27]. After transformation, the cells were incubated in the dark at 28 °C for 12–15 h, and images were taken using a confocal laser scanning microscope (TCS SP8; Leica Microsystems, Wetzlar, Germany).

### 2.5. Histochemical Stain

Leaves from the *OsMed16*-overexpressing plants with obvious lesion mimics and the wild type at the same growth stage were harvested for histochemical analyses. Dead cells were detected by trypan blue staining [28]. The accumulation of H_2_O_2_ was determined using DAB staining [29]. The amount of ROS in cells was determined using NBT staining [30].

### 2.6. RNA-Seq Data Analysis

Three biological replicates of leaves from the *OsMed16*-overexpressing plants (OE-8 line) that displayed spontaneous lesions and wild type plants at the same developmental stage were collected for RNA-Seq analysis. Purification and construction of the cDNA library were performed as previously described [31]. The concentration of the cDNA library is 1.5 ng/μL, and its amount is more than 20 ng. The six RNA-seq libraries were sequenced using the Illumina NovaSeq platform (Illumina, Inc., San Diego, CA, USA) to generate raw reads, and then low quality and adaptor reads were filtered to obtain clean reads for further research.

Sequence reads were aligned to the *Oryza sativa* IRGSP-1 reference genome sequence using HISAT2 v2.0.5. Feature Counts v1.5.0-p3 was used to count the reads numbers mapped to each gene. Additionally, then FPKM of each gene was calculated based on the length of the gene and reads count mapped to this gene. Differential expression analysis of two groups was performed using the DESeq2 R package, and the standards of log2 fold change ≥1 and false discovery rate (FDR) ≤ 0.05 were adopted. To obtain the GO term with significant gene enrichment, GO gene function annotation analysis was performed to obtain functional annotations, biological functions, and metabolic pathways of screened differential genes. Gene Ontology (GO; http://geneontology.org/, accessed on 10 September 2020) analysis of the DEGs was conducted by hypergeometric tests, and each *p*-value indicates the enrichment of the corresponding category.

### 2.7. Phenotypic Analysis of OsMed16 Mutants and OsMed16-Overexpressing Plants

Plants growing in hydroponic media and soil were used for phenotypic observation. The plants were grown hydroponically as described above with 1/2 Kimura B solution (pH 5.6), and the nutrient solution was changed every other day. Soil culture was performed in the rice planting base at Guangxi University. The seedlings growing in the field were covered with a plastic film dome for 30 d to conserve heat. The lesion mimic phenotype was documented when the plants were 48 d old. Agronomic traits, such as effective tillers, seed setting rate, 1000-grain weight, grain width, grain length, and grain number per panicle were analyzed at the mature stage. Each measurement had at least three replicates per sample.

## 3. Results

### 3.1. Sequence and Phylogenic Analysis of OsMed16

**Figure 1:**
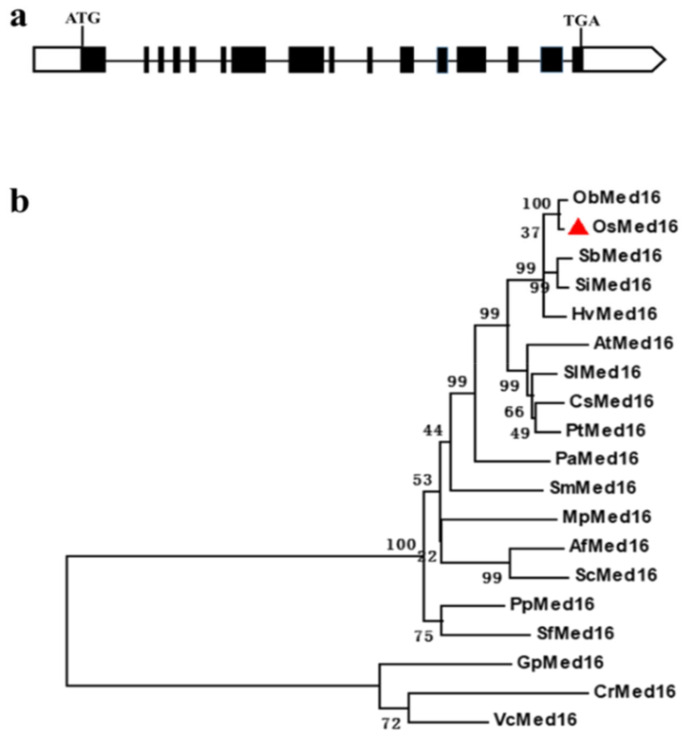
Gene structure and phylogenetic analysis of *OsMed16.* (**a**) Gene structure of *OsMed16*. The boxes (filled and unfilled) represent the exons; the lines between the boxes indicate introns, and the unfilled boxes represent the UTR regions. (**b**) Phylogenetic analysis of *OsMed16* and its counterparts in other plant species. The phylogenetic tree was constructed using the MEGA6 program with the neighbor-joining method. The percentages of replicates in the bootstrap test (1000 replicates) are shown at the branch points of the tree. The first two letters of each protein represent the abbreviated species name. Ob, *Oryza brachyantha*; Os, *O. sativa*; Sb, *Sorghum bicolor*; Si, *Setaria italic*; Hv, *Hordeum vulgare*; At, *Arabidopsis thaliana*; Sl, *Solanum lycopersicum*; Cs, *Cucumis sativus*; Pt, *Populus trichocarpa*; Pa, *Picea abies*; Sm, *Selaginella moellendorffii*; Mp, *Marchantia polymorpha*; Af, *Azolla filiculoides*; Sc, *Salvinia cucullata*; Pp, *Physcomitrella patens*; Sf, *Sphagnum fallax*; Gp, *Gonium pectoral*; Cr, *Chlamydomonas reinhardtii*; Vc, *Volvox carteri.*

Owing to its high homology to *AtSRF6* (*AtMed16*), the rice gene *LOC_Os10g35560* was previously designated as *OsSRF6* [21]. However, as a subunit of the Mediator complex, *LOC_Os10g35560* should be designated as *OsMed16* according to the common unified nomenclature for Mediator subunits [32]. Wathugala et al. [21] predicted that *OsSFR6* (*OsMed16*) encodes a protein of 1170 amino acids. When searching in the GenBank (National Center for Biotechnology Information, NCBI) and Rice Genome Annotation Project databases, we found that the ORF of *OsMed16* was 3906 bp in length and thus encoded a putative protein composed of 1301 amino acid residues, which is 131 aa longer than that of OsSRF6 reported by Wathugala et al. [21]. To test this, the full-length ORF of *OsMed16* (3906 bp) was amplified from the model *japonica* rice variety Nipponbare by high-fidelity PCR and verified by sequencing. The gene structure of *OsMed16* was subsequently analyzed and found to contain 16 exons and 15 introns (Figure 1a).

To understand the evolutionary relationship of *OsMed16*, its counterparts were obtained from different plant species, including algae, mosses, ferns, gymnosperms, and angiosperms. Sequence alignment and phylogenetic analyses were then performed. Overall, the phylogenetic tree is organized into two major clades. The Med16 subunits from unicellular algae (*CrMed16*, *VcMed16*, and *GpMed16*) were grouped into one clade and shared less than 15% identity with OsMed16 (Figure 1b). The Med16 subunits from other plant species were grouped into another clade and shared a higher identity with OsMed16 (Figure 1b). Among the sequences retrieved from NCBI database, OsMed16 displays the highest percentage of identity with ObMed16 from *Oryza brachyantha* (96%) and has 69% identity with AtMed16.

### 3.2. OsMed16 mRNA Expression Pattern and Protein Subcellular Localization

**Figure 2:**
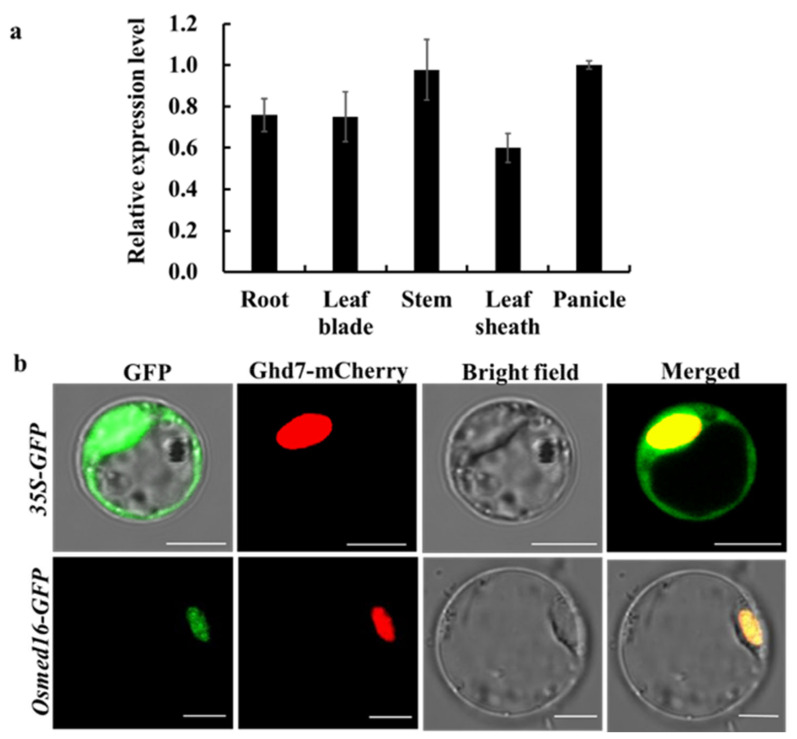
Organ-specific expression and subcellular localization of *OsMed16*. (**a**) Expression of *OsMed16* in different rice organs analyzed by qRT-PCR. Data are the means ± SD of three biological replicates. (**b**) Subcellular localization of OsMed16. GFP: OsMed16 or GFP was transiently expressed in rice protoplasts with Ghd7-mCherry. Fluorescence signals from GFP, mCherry, and the merged images are shown. Free GFP was used as a control. Bars = 10 μm.

Quantitative real-time PCR (qRT-PCR) assays were performed with total RNA isolated from rice roots, leaves, stems, leaf sheaths, and young panicles. The results showed that *OsMed16* mRNA was expressed in all the examined organs except leaf sheaths which had a lower level of expression (Figure 2a). Furthermore, public microarray databases, such as the eFP browser, indicated that *OsMed16* was also expressed in inflorescences and seeds (Figure S1) [33]. The wide pattern of expression of *OsMed16* is consistent with its function as a basic transcriptional regulator.

To determine the subcellular localization of OsMed16, a p35S-*OsMed16***-***GFP* construct was generated and transiently expressed in rice protoplasts with a red fluorescent protein (RFP) fused to OsGhd7, a protein localized to the nucleus [34]. The p35S-GFP empty vector was used as a control. As a result, the green fluorescence signal in the control was observed in the cytoplasm, while fluorescence from OsMed16-GFP was present in the nucleus and colocalized with the OsGhd7-RFP protein (Figure 2b). These results indicated that OsMed16 is localized in the nucleus, which is consistent with its role as a Mediator subunit.

### 3.3. Overexpression of OsMed16 Caused the Inhibition of Growth of Rice and Spontaneous Cell Death

To investigate the function of *OsMed16* in planta, the gene was disrupted using CRISPR/Cas9 genome-editing technology (Figure S2a). The *osmed16* mutants exhibited a stunted growth phenotype, failed to head, and died prematurely (Figure S2b), indicating that disruption of *OsMed16* caused lethality in rice seedlings.

**Figure 3:**
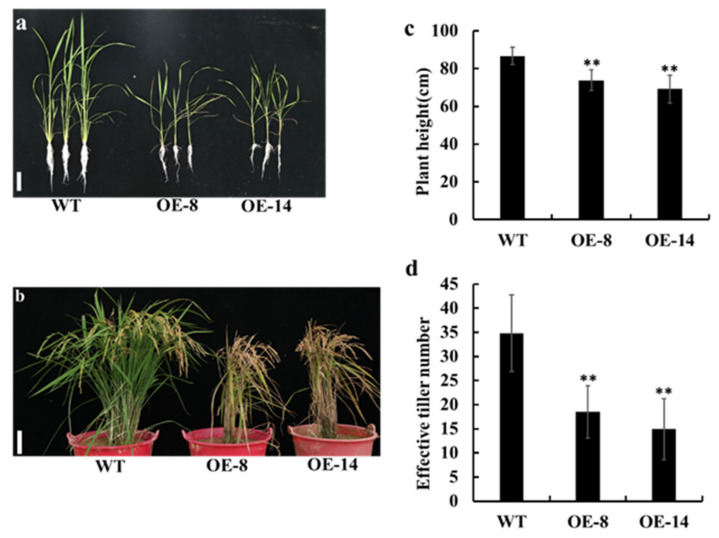
*OsMed16* overexpression inhibited rice growth. (**a**) Phenotypes of the *OsMed16*-overexpressing (OE) and wild type (WT) rice seedlings. Scale bars = 10 cm. (**b**) Phenotypes of the OE and WT rice plants at the mature stage. Scale bars = 10 cm. (**c**,**d**) Comparison of plant height (**c**) and tiller number (**d**) of the WT and OE plants at mature stage. Values are the mean ± SD (*n* = 10). Asterisks indicate significant differences from the wild type (** *p* < 0.01 using Student’s *t*-test).

**Figure 4:**
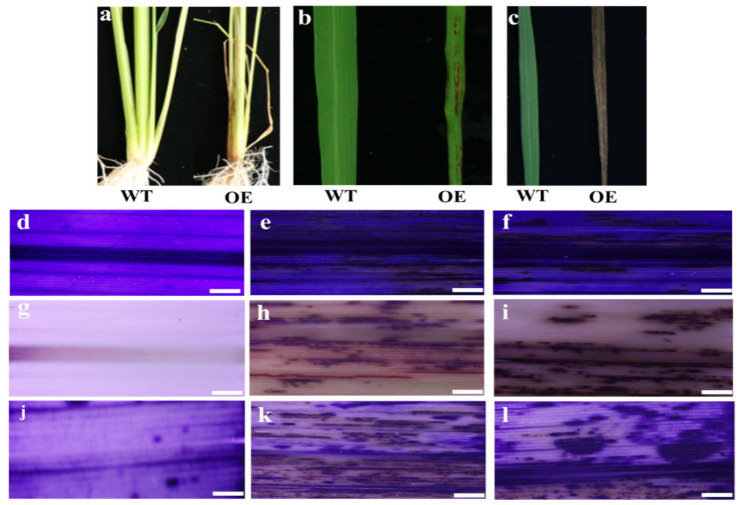
Overexpression of *OsMed16* caused spontaneous cell death. (**a**–**c**) Lesion phenotype in leaf sheath and flag leaf of *OsMed16*-OE plants grown for 30 (**b**) and 60 d (**c**). Trypan blue staining (**d**–**f**), DAB staining (**g**–**i**) and NBT staining (**j**–**l**) of the leaves of the wild type and *OsMed16*-OE plants grown in a nutrient solution for 48 d. WT (**d**,**g**,**j**), OE-8 (**e**,**h**,**k**), OE-14 (**f**,**i**,**l**). Scale bars = 2 mm.

We further employed a gain-of-function approach to investigate the roles of *OsMed16*. The overexpression vector of *OsMed16* driven by a maize ubiquitin promoter was constructed and transformed into Nipponbare using an *Agrobacterium*-mediated method. The level of expression of *OsMed16* in the transgenic plants was detected using a qRT-PCR assay, and two representative homozygous transgenic lines with high levels of expression of *OsMed16* (designated *OsMed16*-OE) were used for further investigation (Figure S3). Unexpectedly, the overexpression of *OsMed16* also inhibited the growth of rice. Compared with the wild type, *OsMed16*-OE lines had a dwarf phenotype with fewer tillers (Figure 3d). Another distinct visible phenotype observed was spontaneous cell death in the *OsMed16*-OE lines. Small necrotic spots first appeared on the leaf sheath of *OsMed16*-OE seedlings at the three-leaf stage (Figure 4a) and were also observed on leaves (spotted leaf, Figure 4b,c). As the plants grew, the brown spots gradually became large irregular lesions (Figure 4b,c). The cell death was further confirmed using Trypan Blue staining. The *OsMed16*-OE leaves had an increased intensity of staining compared with the wild type leaves (Figure 4d–f). The accumulation of reactive oxygen species (ROS) may cause cell damage and even death [35]. Overaccumulation of H_2_O_2_ was observed in the leaves of *OsMed16-*OE plants using 3,3′-diaminobenzidine (DAB) staining (Figure 4g–i). We also used nitroblue tetrazolium (NBT) staining and observed an increase in superoxide anions in *OsMed16-*OE plants (Figure 4j–l). With the increase in the number and size of lesions, the old leaves of *OsMed16*-OE lines withered prematurely, and the whole plants exhibited early senescence (Figure 3b).

### 3.4. Overexpression of OsMed16 Reduced the Yield of Rice Grains

**Figure 5:**
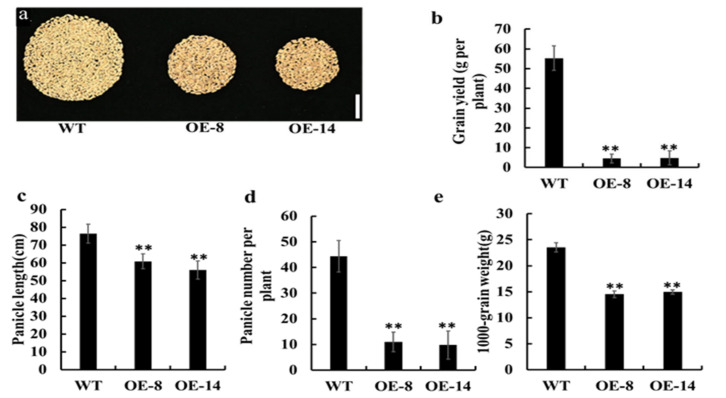
Overexpression of *OsMed16* reduced rice grain yield. (**a**) Total grains per plant of WT and *OsMed16*-OE plants grown in the field. Scale bars = 10 cm. (**b**–**e**) Comparison of grain yield per plant (**b**), panicle length (**c**), panicle number per plant (**d**) and 1000-grain weight (**e**) of the WT and *OsMed16*-OE plants. Data (**b**–**e**) are the means ± SD of three biological replicates. WT, wild type. ** *p* < 0.01.

In addition to the inhibition of growth, plants overexpressing *OsMed16* also exhibited a significant reduction in yield. Compared with the wild type plants, the yield of grain per plant was reduced by 91.8% and 91.3% in the two overexpression lines (Figure 5a,b). The components of yield were analyzed in more detail. The panicle number per plant, panicle length, and 1000-grain weight of the *OsMed16*-OE plants decreased significantly compared with those of the wild type (Figure 5c–e). Additionally, the seed length and width were also compared between the *OsMed16*-OE lines and the wild type. The results showed that the seed length was unchanged (Figure S4a,b), but the seed width decreased slightly in the *OsMed16*-OE lines (Figure S4c,d).

### 3.5. Transcriptome Changes in OsMed16-OE Plants

**Figure 6:**
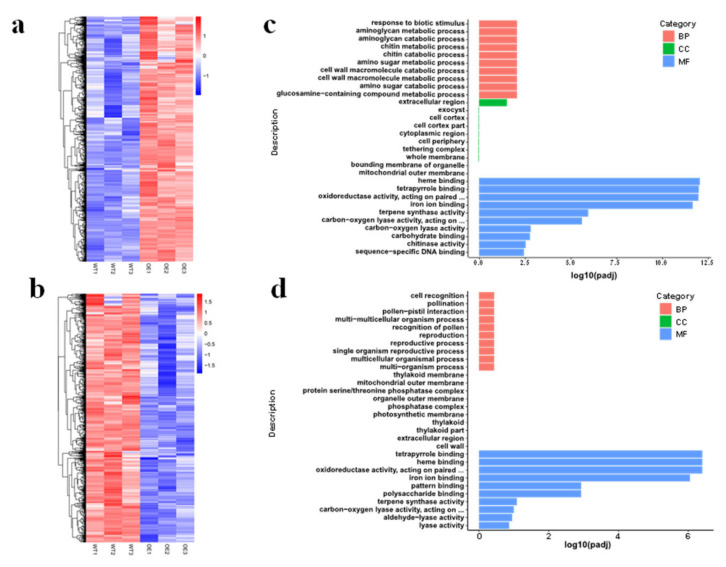
Heatmap and GO enrichment analysis of DEGs between *OsMed16*-OE and wild type plants. (**a**) Compared with the wild type, 1419 upregulated genes were shown as Heatmap. (**b**) Compared with the wild type, 983 downregulated genes were shown as Heatmap. (**c**) Differentially upregulated gene GO rich map in the WT and OE leaves with lesions. (**d**) Differentially downregulated gene GO rich map in WT and OE leaves with lesions. DEGs, differentially expressed genes; GO, gene ontology; WT, wild type. |log2 fold change| ≥ 1 and *p*-value (false discovery rate, FDR) ≤ 0.05.

To assess the influence of *OsMed16* overexpression on gene expression, *OsMed16-*OE plants that exhibited necrotic lesions were harvested, and RNA sequencing (RNA-Seq) was performed on the wild type and *OsMed16-*OE plants. Overall, we obtained six transcriptome data sets, with each containing an average of approximately 50 million paired-end (PE) reads (Figure S5). The raw sequencing reads were first trimmed and mapped to the rice reference genome using HISAT2. More than 96% of the reads were mapped to unique loci per sample (Figure S5). Differentially expressed genes (DEGs) were determined with stringent criteria: |log_2_ fold change| ≥ 1 and *p*-value (false discovery rate, FDR) ≤0.05. Compared with the wild type, 2402 DEGs were detected in *OsMed16-*OE plant leaves, of which 1419 were upregulated (Figure 6a, Table S2), whereas 983 were downregulated (Figure 6b, Table S3). Gene ontology (GO) enrichment analysis indicated that the upregulated genes in *OsMed16-OE* plants were involved in multiple biological processes, including the binding of heme (66) and tetrapyrrole (66), oxidoreductase activity (57), and the binding of iron ions (56). Among these DEGs, *CYP71Z2* (*LOC_Os07g11739*) is a rice cytochrome P450 gene and participates in plant defense by regulating the secondary metabolism of a phytoalexin [36,37]. The rice *D3* gene (*LOC_Os06g06050*), a multitiller dwarf gene, encodes an F-box protein rich in leucine repeat sequences, which is not only necessary for the signal transduction of strigolactone (SL) but is also involved in leaf senescence and cell death [38]. *OsCAld5H1* (*LOC_Os10g36848*) encodes a ferulic acid 5-hydroxylase, whose biological function is primarily involved in the synthesis of rice lignin, and its expression affects the composition of S/G lignin in the main nutritional tissues of rice without affecting the structure of vascular bundles [39]. *HAN1* (*LOC_Os11g29290*) encodes an oxidase that can catalyze the conversion of biologically active jasmonate-L-isoleucine (JA-Ile) into the inactive 12-hydroxy-jasmony-L-isoleucine (12OH-Ja-ILE) and regulate JA-mediated low temperature reaction and cold tolerance as a negative regulator of cold tolerance [40]. In contrast, the downregulated genes were mapped to categories including tetrapyrrole binding (40), heme binding (39), and oxidoreductase activity (34) (Figure 6a,b). Among these genes, *OsAPX2* (*LOC_Os07g49400*) is an ascorbic acid peroxidase gene that plays an important role in the growth and development of rice by clearing ROS to protect the seedlings from abiotic stress [41]. *CYP93G2* (*LOC_Os06g01250*) encodes flavanone 2-hydroxylase, which is not only a cytochrome P450 gene but also the first enzyme in its biosynthetic pathway [42]. Our results confirmed that the upregulated and downregulated genes were indeed associated with multiple biological pathways in rice.

**Figure 7:**
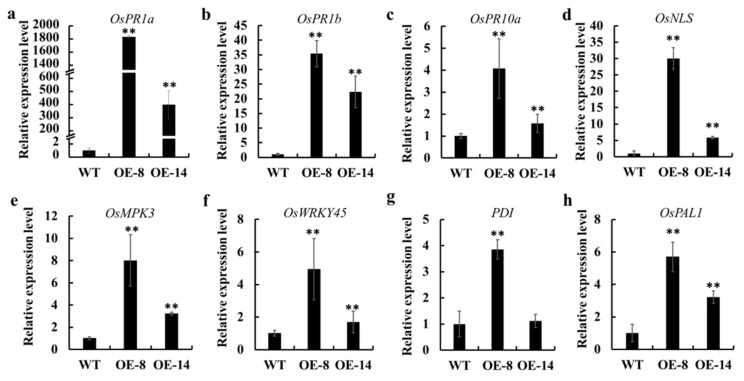
Elevated expression of some defense-related genes in *OsMed*16-OE plants. The patterns of expression of *OsPR1a* (**a**), *OsPR1b* (**b**), *OsPR10a* (**c**), *OsNLS* (**d**), *OsMPK3* (**e**), *OsWRKY45* (**f**), *PDI* (**g**), and *OsPAL1* (**h**) genes in OE and wild type (WT); data are the means ± SD of three biological replicates. ** *p* < 0.01.

The overexpression of *OsMed16* led to spontaneous cell death in rice, which resembled the hypersensitive response (HR) caused by pathogenic infection. This led us to hypothesize that the overexpression of *OsMed16* could trigger the expression of defense-related genes. Thus, we examined these genes in the RNA-Seq data. Indeed, some defense-related genes, including *PR1a* and *PR1b*, were upregulated in *OsMed16*-OE compared with those in wild type. To confirm these results, we then performed qRT-PCR to examine the levels of expression of eight defense-related genes in the *OsMed16*-OE and wild type plants. These genes, including *OsPR1a*, *OsPR1b*, *OsPR10a*, *OsNLS*, have been reported to participate in rice defense responses in the previous studies [43,44,45]. The transcript levels of all these genes were elevated in the *OsMed16*-OE plants (Figure 7), suggesting that the overexpression of *OsMed16* activated the expression of defense-related genes.

## 4. Discussion

To date, the research on the plant Mediator complex subunit Med16 has primarily focused on *Arabidopsis thaliana*, while less research has been conducted on rice. Wathugala et al. [21] reported that when the *OsMed16*/*OsSFR6* gene was ectopically expressed in the *Arabidopsis* mutant *atsrf16*, it restored the wild type phenotype of the *atsrf16* mutant. The main function of the *OsMed16* gene in rice has not been reported. In this study, *OsMed16* was identified in rice, and the *OsMed16* gene knockout mutants and overexpression lines were constructed for the first time to our knowledge. The function of the Mediator complex subunit Med16 in rice was studied using reverse genetics. The results showed that the leaves of plants overexpressing *OsMed16* exhibited brown spots, and the spots continued to expand with the growth of the plant until they filled the entire leaf, resembling spontaneous lesions.

Spontaneous lesions refer to lesion-like spots that are spontaneously produced on the leaf surface without pathogen infections. They are very similar to the lesions associated with HRs triggered by incompatible pathogens [46]. Spontaneous lesions are widely present in various plants, including *Arabidopsis*, rice, corn, wheat, barley, and soybeans [47,48,49,50,51,52,53]. In this study, the pathological changes of *OsMed16*-overexpressing plants were primarily caused by the abnormal expression of *OsMed16*. The plants overexpressing *OsMed16* spontaneously displayed necrotic spots in the absence of pathogens. Simultaneously, the expression levels of defense-related genes *PR1* and *PR10a* [52] were upregulated in overexpressing plants, suggesting that *OsMed16* may exist in a signaling pathway that is usually activated in the absence of pathogen infection, leading to hypersensitivity in *OsMed16*-overexpressing plants. Moreover, we searched the genes coexpressed with *AtMed16* in *Arabidopsis* using Botany Array Resource (BAR) Expression Angler [54]. Some disease resistance genes such as At5g38340 were identified, suggesting that *Med16* coexpressed with the defense-related genes in both monocots and dicots. The cell death phenotype and activation of defense-related genes in *OsMed16*-overexpressing plants indicated that *OsMed16* may play a positive regulatory role in programmed cell death (PCD) and resistance-related signaling pathways in plants. However, the mechanism of the positive regulation of PCD and defense signals by the *OsMed16* gene is still unclear. The results of this study will provide a new perspective for the molecular regulatory mechanism of the Mediator complex in plant cell death and disease resistance signaling, particularly in monocotyledons. The results of analysis of agronomic traits showed that the overexpression of *OsMed16* seriously affected the growth and development of plants. Therefore, it is hypothesized that *OsMed16* may be related to the overall physiology and morphology of plants. Further elucidating the mechanism of *OsMed16* and its downstream, genes will contribute to the understanding of role of *OsMed16* in the regulation of plant cell death and defense mechanisms, as well as growth and development of the rice plant.

As part of general transcriptional regulation, the Mediator complex subunits connect specific transcriptional activators to the RNA polymerase II complex, and some of the individual Mediator complex subunits receive signals from specific pathways and transfer them to general transcriptional mechanisms. Some Mediator complex subunits have been shown to be related to defense, such as Med21 and Med25 [14,55,56,57,58,59]. Moreover, in *A. thaliana*, Med16 was not only proven to regulate the immune response but also proven to be an important subunit in the tail module, as the whole tail module was missing after extraction of the mediator complex in *atsrf6* mutants. Our experimental results showed that the *OsMed16*-overexpressing plants affected the various periods of rice growth and development—i.e., *OsMed16* plays a crucial role in different developmental stages. However, the mechanisms by which *OsMed16* regulates the growth and development of rice are still largely unknown. Future studies will be required to dissect these regulatory mechanisms.

RNA-seq technology was used to analyze the transcriptome of the *OsMed16*-overexpressing plants and wild type plants in this study. Compared with the wild type, we detect 2402 DEGs in *OsMed16*-OE plant leaves. GO enrichment analysis showed that the most differentially expressed genes involved in molecular function, which can be attributed to binding and catalytic genes. These results indicate that differentially expressed genes are mainly involved in cell and metabolic processes and are active in these two processes. Many of these differential genes are related to plant development, which indicates that *OsMed16*-overexpression does affect the growth and development of rice.

## 5. Conclusions

In this study, the rice Mediator subunit 16, *OsMed16*, was functionally characterized. It is expressed in various rice organs and localized in the nucleus. The loss of function of *OsMed16* causes rice seedling lethality. Its overexpression led to the inhibition of rice growth, low yield, and spontaneous cell death in the leaf blades and sheaths. RNA-Seq data suggested that the overexpression of *OsMed16* altered the expression of a large number of genes, including a number of defense-related genes. These results demonstrated that *OsMed16* regulates not only rice growth and development but also the defense response.

## Data Availability

All the data supporting the conclusions of this article are provided within the article and in its additional files. All data and materials are available upon reasonable request from the corresponding author.

## Supplementary Materials

The following are available online at https://www.mdpi.com/article/10.3390/genes12050656/s1, Figure S1: Transcript level of *OsMed16* in the developing inflorescence and seed, Figure S2: Knockout of *OsMed16* using CRISPR technology that causes rice seedling lethality, Figure S3: Detection of the levels of expression of *OsMed16* in wild type and transgenic plants using qRT-PCR, Figure S4: Grain shape of *OsMed16* overexpression plants, Figure S5: Basic statistics of the RNA-Seq data, Table S1: Primers used for defense-related genes, Table S2: Compared with the wildtype, 1419 genes were upregulated in OsMed16-OE plant leaves, Table S3: Compared with the wild type, 983 genes were downregulated in OsMed16-OE plant leaves.
