# Supplementary material for: Overexpression of OsMed16 Inhibits the Growth of Rice and Causes Spontaneous Cell Death
Source: Genes (Basel). 2021 Apr 27;12(5):656. doi: 10.3390/genes12050656 (PMC8145620; doi:10.3390/genes12050656)
Supplement: Supplementary file 1 [file genes-12-00656-s001.zip › Supplementary materials-Genes.pdf]

Supplementary Materials

Overexpression of *OsMed16* inhibits the growth of rice and causes spontaneous cell death

Jie Jiang[1 #], Guangzhe Yang[1 #], Yafeng Xin[1], Zhigang Wang[1], Wei Yan[2,3], Zhufeng Chen[3,4], Xiaoyan Tang[2,3 *], Jixing Xia[1*]

[1] *State Key Laboratory for Conservation and Utilization of Subtropical Agro-bioresources, College of Life Science and Technology, Guangxi University, Nanning 530004, China*

[2] *Guangdong Provincial Key Laboratory of Biotechnology for Plant Development, School of Life Sciences, South China Normal University, Guangzhou 510631, China*

[3] *Shenzhen Institute of Molecular Crop Design, Shenzhen 518107, China*

[4] *Shenzhen Agricultural Technology Promotion Center，Shenzhen 518055, China*

# These authors contributed equally to this work.

* Corresponding author: txy@frontierag.com (X.T.); xiajx@gxu.edu.cn (J.X.)

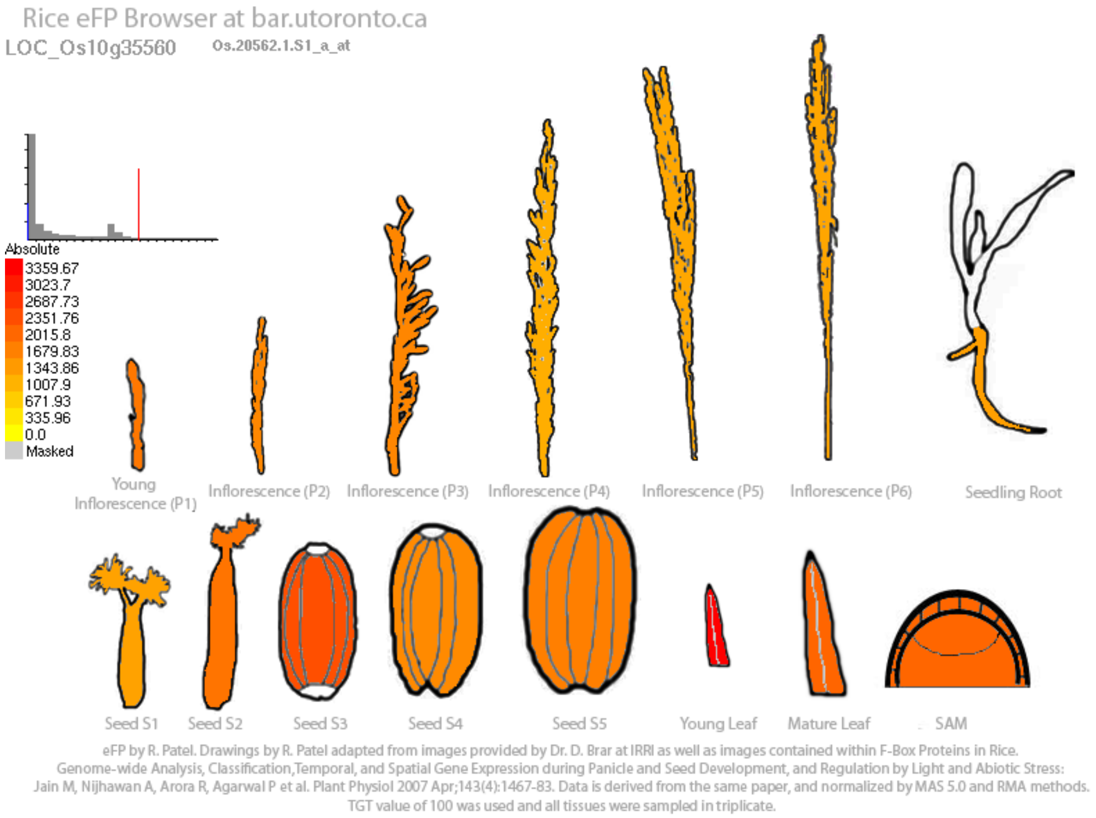

Figure S1. Transcript level of *OsMed16* in the developing inflorescence and seed.

The result was extracted from the eFP browser database [31].

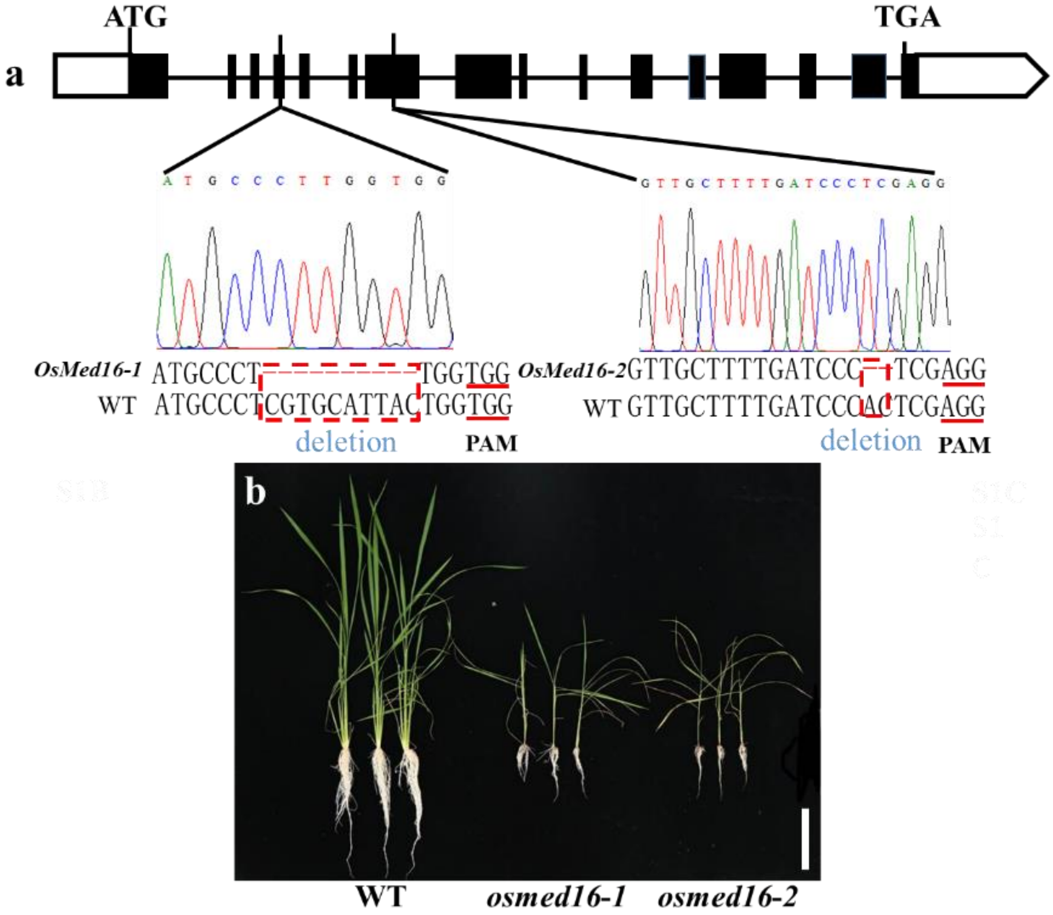

Figure S2. Knockout of *OsMed16* using CRISPR technology that causes rice seedling lethality. (a) The two target site mutations of *OsMed16* gene. The black inverted triangle indicates the positions of the two targets; The red dashed box is positioned at the mutation site of the mutants. (**b)** The phenotype of the wild type and two *OsMed16* knockout lines of 48-d-old seedlings. Scale bars = 10 cm. WT, wild type.

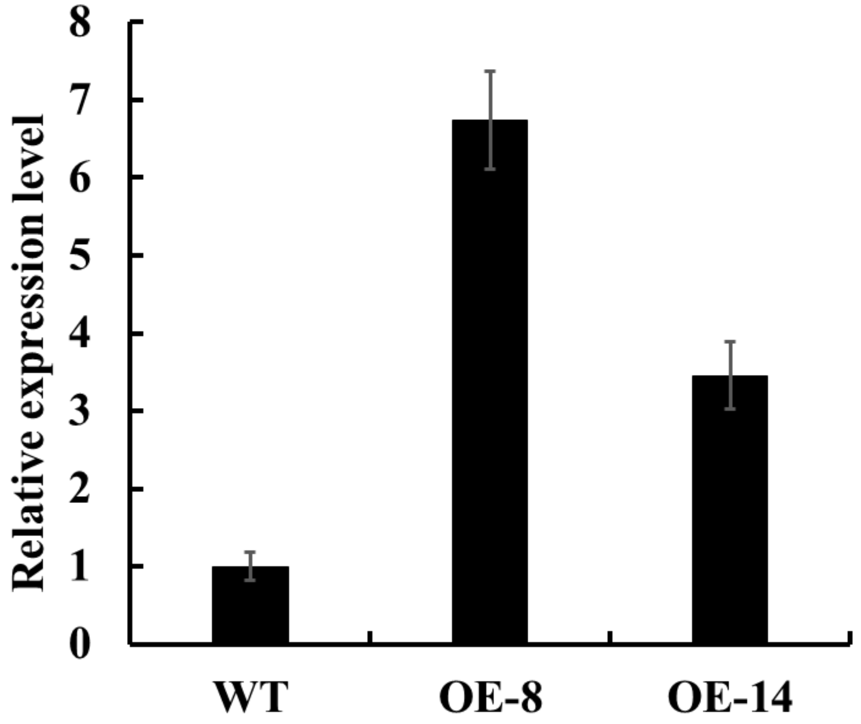

Figure S3. Detection of the levels of expression of *OsMed16* in wild type and transgenic plants using qRT-PCR. RNA was extracted from the roots of seedlings. The expression of rice *ACTIN* was used as the internal control. qRT-PCR, quantitative real time PCR; WT, wild type.

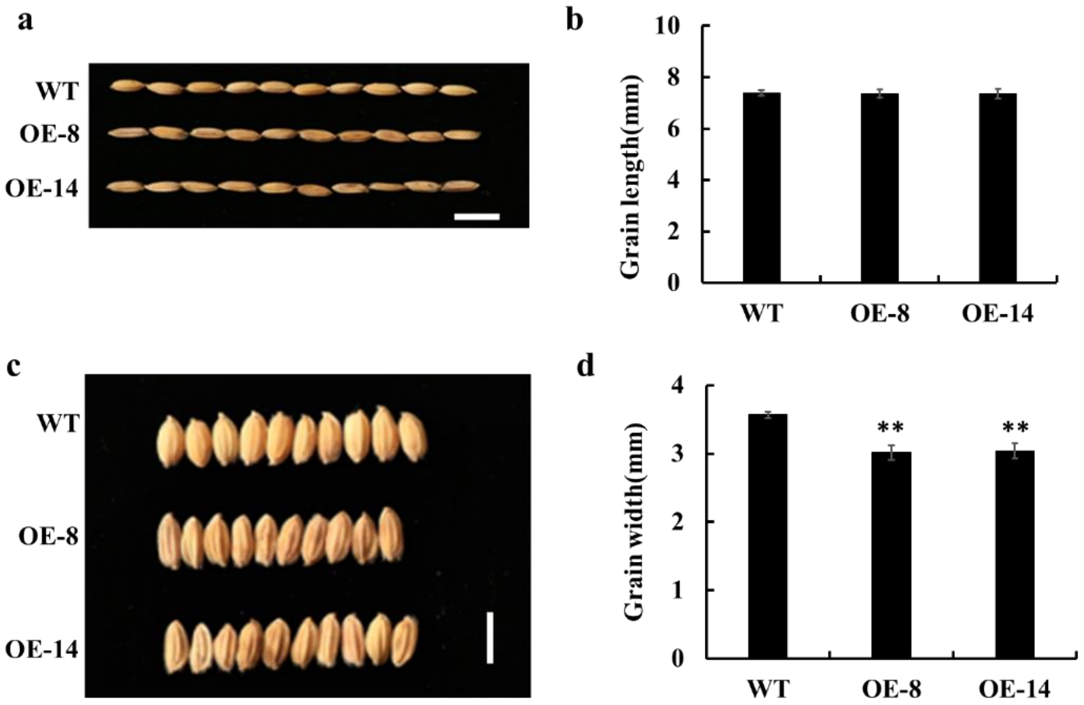

Figure S4. Grain shape of *OsMed16* overexpression plants. Comparisons of grain length (**a** and **b**) and grain width (**c** and **d**) between the wild type and *OsMed16*-OE plants. Scale bars = 5 mm. Data (**c** and **d**) are the means ± SD of three biological replicates. *P < 0.05. * *P < 0.01.

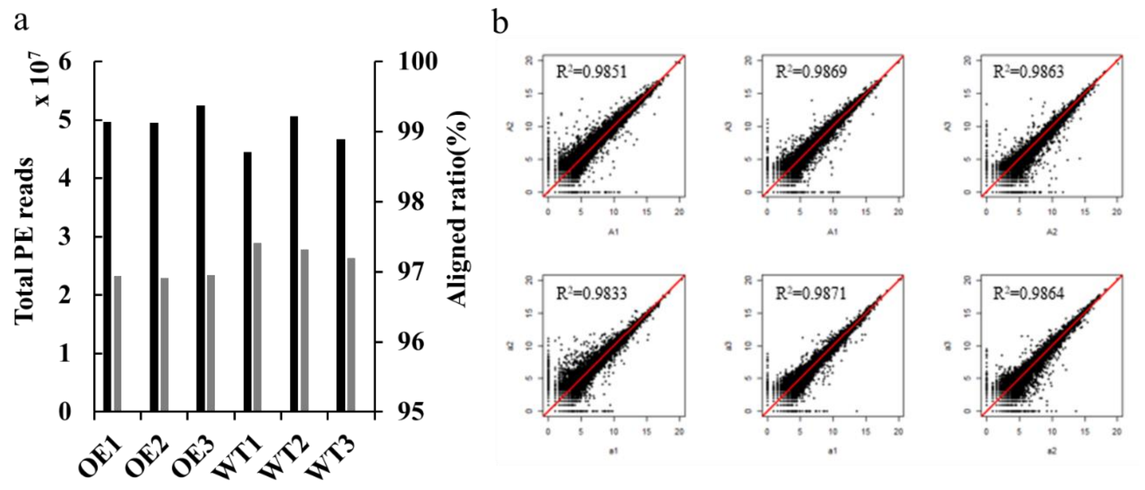

Figure S5. Basic statistics of the RNA-Seq data. (**a**) Statistics of total paired-end reads (black bar) and aligned ratio (gray bar) for each replicate. (**b)** Consistency of samples and replicates.

Table S1. Primers used for defense-related genes.

Gene name	Forward primer (5'-3')	Reverse primer (5'-3')
OsPR1a	CGTGTCGGCGTGGGTGT	GGCGAGTAGTTGCAGGTGATG
OsPR1b	TACGCCAGCCAGAGAAGC	GCCGAACCCCAGAAGAGG
OsPR10a	GTCCGGGCACCATCTACACC	CAAGCTTCGTCTCCGTCGAGT
OsNLS	GAAGACCACCCTTGCCAATCA	GCTTGTTGATGATGGCTTGC
OsMPK3	CACCTCGACCACGAGAACATCA	GGCAGTGCTCTTCTGACAGTTC
OsWRKY45	GCCGACGACCAGCACGATCACC	AGGCTGCTCAGCACCTCCTCCT
PDI	CGTCGAGTTCTACGCCCCGT	CCTGAATGTTCTTGCCCTGGTTCC
OsPAL1	TTCGACGCCAACATCCTCG	GCTTCAGCTTGTGGGTCAGGT
